# Delayed Therapy of Descending Aortic Coarctation Results in Anterior Cerebral Rupture: A Case Report

**DOI:** 10.3389/fped.2021.654705

**Published:** 2021-10-04

**Authors:** Kele Qin, Jinfu Yang, Mi Tang, Chukwuemeka Daniel Iroegbu, Shijun Hu, Chengming Fan

**Affiliations:** Department of Cardiovascular Surgery, The Second Xiangya Hospital, Central South University, Changsha, China

**Keywords:** descending aortic coarctation, delayed therapy, rupture, congenital heart disease, surgery

## Abstract

**Background:** Coarctation of the aorta (CoA) is the congenital constriction or narrowing of the aortic lumen. These constrictions are primarily located in the descending aorta causing significant discrepancies in systolic blood pressures of the upper and lower extremities. Thus, a delay in diagnosis and treatment may lead to severe and adverse consequences.

**Case presentation:** Herein, we present a 13-year-old boy with anterior cerebral rupture following a delayed diagnosis for descending CoA. Percutaneous transluminal balloon dilatation and endovascular stent implantation were urgently and successfully performed alongside cerebral clipping of the vascular aneurysm.

**Conclusion:** An early diagnosis is crucial for CoA's successful treatment and management to prevent complications, including anterior cerebral rupture.

## Background

Coarctation of the aorta (CoA) is one of the most common congenital heart disease malformations and represents a spectrum of aortic narrowing that varies from a discrete entity to tubular hypoplasia (1). Notably, if thorough physical examinations are not performed, patients with this congenital cardiac malformation can survive for an extended period, given the subtle clinical signs associated with CoA (2). Interestingly, CoA is also a secondary cause of hypertension (2, 3). In a facility-based review of cases reported by Woldmichael and Aklilu delayed diagnosis coupled with a delay in intervention after diagnosis were found in some CoA patients (4).

Clinically, CoA is a congenital cardiac malformation that is often misdiagnosed despite specific physical findings (5); therefore, early diagnosis and referrals for patients with CoA are recommended (4). The importance of upper and lower extremity blood pressure determination is emphasized as part of an initial routine physical examination (5). Thus, a delay in diagnosis and treatment may cause severe and adverse consequences. Also, long-term complications without timely intervention result in refractory hypertension, including premature coronary artery disease, stroke, endocarditis, aortic dissection, and heart failure (6).

We present a 13-year-old boy with an anterior cerebral rupture following a delayed diagnosis for descending CoA. Percutaneous transluminal balloon dilatation and endovascular stent implantation were urgently and successfully performed alongside cerebral clipping of the vascular aneurysm.

## Case Presentation

A 13-year-old boy was referred to the local hospital with a 6-month history of headache and dizziness with no obvious predisposing causes. He denied any familiar history of hypertension, psychosocial history including genetic information, or other cardiac health comorbidities. Nonetheless, the local physician prescribed Amlodipine (25 mg, qd) for hypertension treatment. The patient was eventually transferred to our center with an explosive onset of headache, vomiting, dyspnea, and fatigue. Physical examination showed normal body development. The upper and lower limbs' blood pressure was 200/130 and 98/78 mmHg, respectively. A heart rate of 85 bpm with significant pulsation in the suprasternal fossa was detected. A systolic murmur was audible at the paravertebral area. Radial pulse was palpable, whereas the dorsal pedal artery was not.

**Figure 1:**
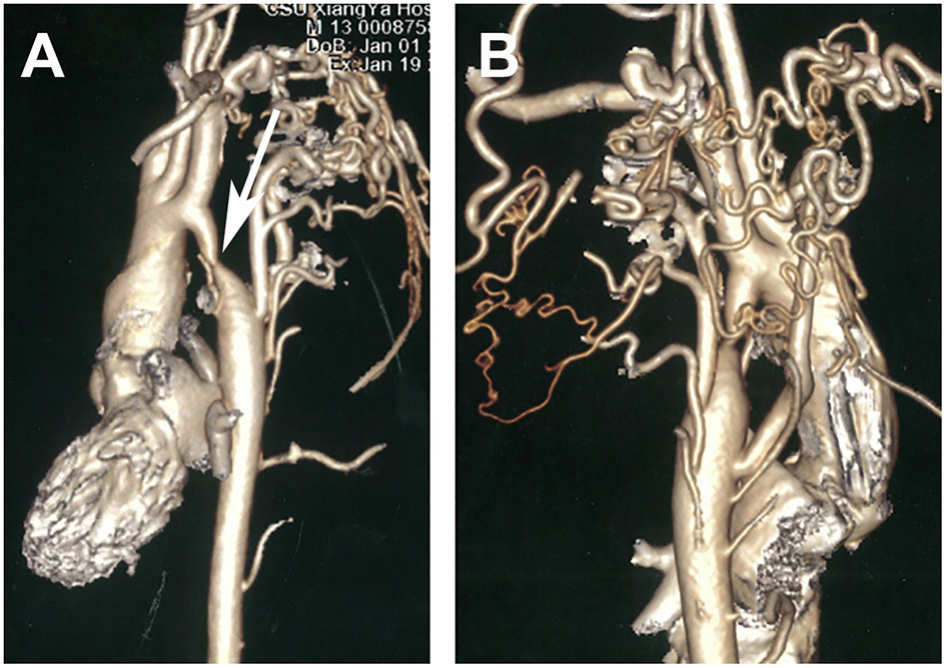
Computed tomography angiography preoperatively showing a coarctate descending aorta [(**A)**, arrow] with more than eight collateral vessels between the proximal and distal coarctated tissue **(B)**.

**Figure 2:**
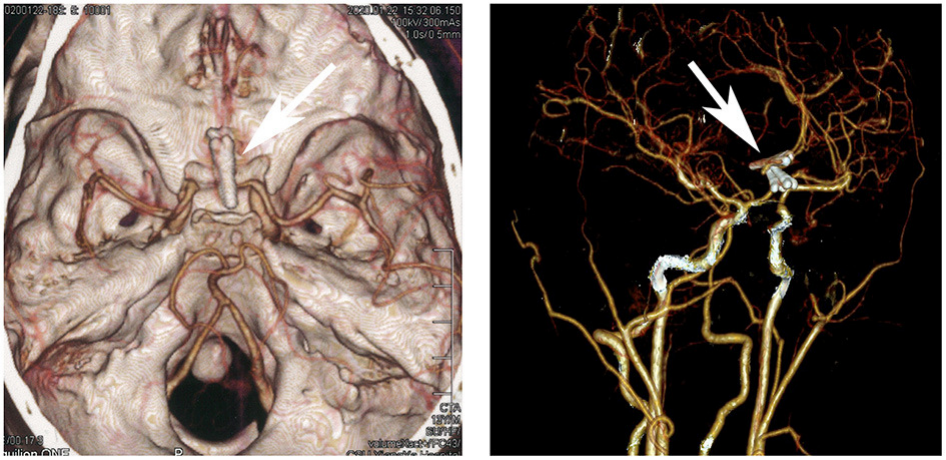
Cerebral computed tomography angiography showing the ruptured anterior cerebral aneurysm (arrow).

**Figure 3:**
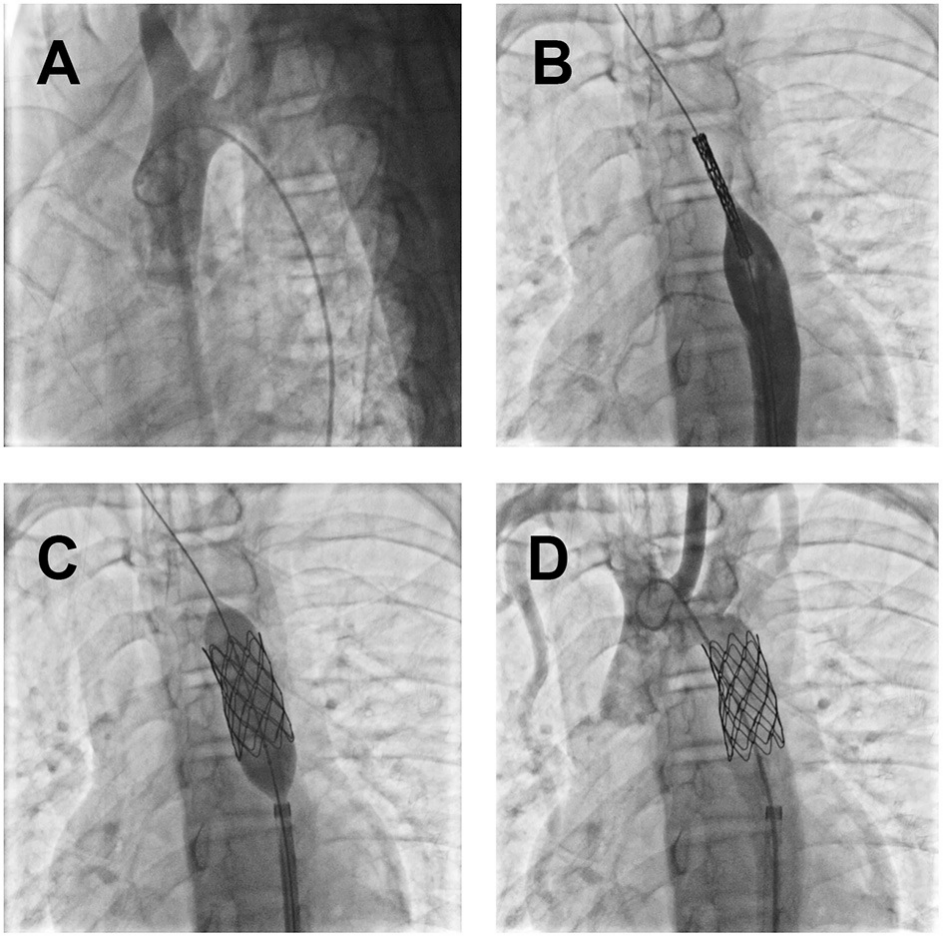
Coarctation intervention with the guidance of digital subtraction angiography: **(A)** The coarctated descending aorta was confirmed by the poor perfusion of the iodine contrast medium to the distal segment; **(B)** inserted endovascular stent; **(C)** balloon dilatation and endovascular stent implantation in the constricted descending aorta; **(D)** a free flow of the perfused contrast medium to the distal segment just after the operation.

Electrocardiogram results showed sinus rhythm, incomplete right bundle branch block, and potential ventricular hypertrophy. Chest X-ray showed no remarkable findings with a standard cardiothoracic index. However, transthoracic echocardiography showed a peak systolic gradient of 64 mmHg at the coarctation site of the descending aorta. A hypoplastic artery was also noted distally to the coarctation (13 mm). Cerebral computed tomography scan and angiography (CTA) were performed to detect the cause of headaches and dizziness. The results indicated an aneurysmal rupture of the anterior cerebral artery. A cardiac CT scan was further performed, and a 3 mm coarctated descending aorta (Figure 1A, arrow), with over eight collateral vessels between the proximal and radial coarctated tissue (Figure 1B) was revealed. An aneurysmal clip (Figure 2, arrow) was successfully applied, followed by balloon angioplasty and endovascular stent implantation in the coarctated site of the descending aorta (Figure 3).

**Figure 4:**
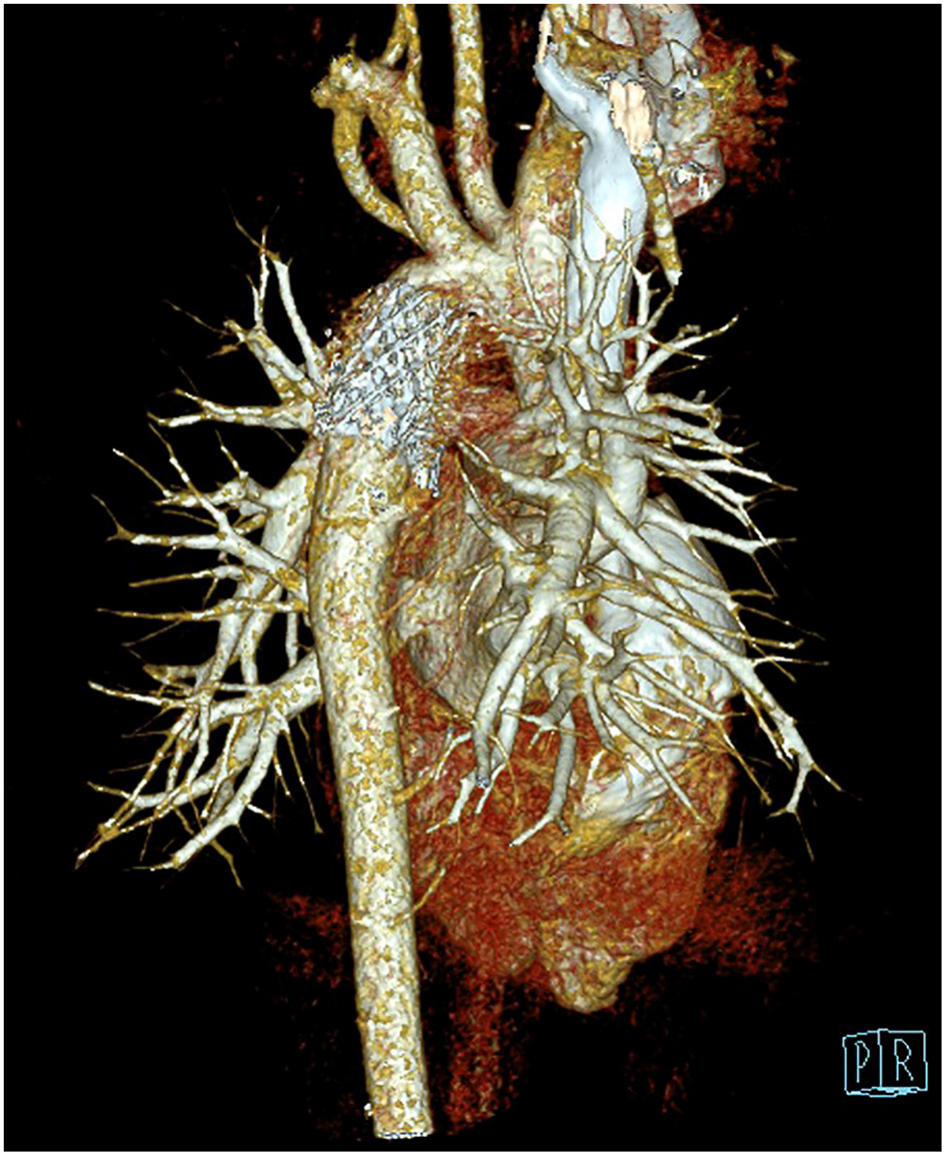
Computed tomography angiography postoperatively showing the coarctated descending aorta after endovascular stent dilatation.

The postoperative outcomes were satisfactory. The blood pressure was also significantly alleviated on both upper and lower limbs. Systolic and diastolic blood pressures were at 116/77 and 123/79 mmHg, respectively. The constricted segment of descending aorta was dilated up to 16 mm, which was detected by the chest CTA 3 days after the endovascular procedure (Figure 4). The patient recovered without complications and was discharged on the seventh post-operative day in a stable condition with a recommendation of subsequent follow-up.

## Discussion and Conclusions

CoA, a fundamental cause of secondary hypertension and discrepant upper and lower extremity pulse, is a treatable congenital malformation. Patients often complain of headaches, leg fatigue, or claudication (7). The prevalence of CoA varies from 5 to 8% of all congenital heart defects and is only occasionally diagnosed in adults (8). A study by Woldmichael and Aklilu showed that postoperative survival was much higher in younger patients and declined with advanced age (4). Without therapeutic intervention, the mean life expectancy for patients with CoA is 35-years old, and ~90% of these patients die before 50-years of age (7).

Severe discrepant hypertension, aortic dissection, stroke, and congestive heart failure are common complications in patients without timely therapeutic intervention or delayed therapy (7). Optional therapeutic strategies, including open chest surgery, percutaneous balloon dilatation, or stent implantation, primarily rely on the patient's age, preference, associated lesions, and the team's experience. In asymptomatic patients, the repair is recommended between 2 and 5-years of age (8). The degree and duration of hypertension before the intervention and the patient's age may significantly affect long-term survival. Hence, the treatment goal for most coarctation is to improve distal perfusion and control hypertension by surgical correction, balloon angioplasty, or stent implantation (9). Thus, a delay in diagnoses and CoA treatment would cause severe secondary upper body hypertension, which may further cause aneurysm or stroke.

In addition, a prolonged afterload increase in the left ventricle would accelerate ventricular remodeling and heart failure. Thus, early diagnosis and CoA treatment are of the utmost importance. In the study by Rao (8), an experienced cardiologist applied balloon angioplasty to relieve the obstruction, but concerns for aneurysms and arterial complications lingered. In the present study, corrective treatment was deemed mandatory, considering the high aortic gradient caused by the coarctated lumen, alongside concomitant hypertension and anterior cerebral rupture. However, given the patients' preferred choice of therapeutic management, a group of cardiac surgeons performed balloon angioplasty combined with endovascular stent implantation. The procedure was performed in a hybrid operating room in case urgent thoracic surgery is required. Although the short-term results for surgical or balloon therapy for isolated coarctation are favorable, long-term following-up is needed.

The patient in the case herein was diagnosed with CoA. The craniocerebral aneurysm might have resulted from the prolonged and recurrent hypertension secondary to the constricted descending aorta. It is pivotal to note that crucial aspects of the present study were limited, given that CoA patients are predisposed to intracranial aneurysms (IAs) and asymptomatic until rupture occurs, with an overall mortality rate of ≈45% (9). Approximately 30% of survivors have moderate to severe disabilities, and 66% (with “successful” clip placements) improved overall quality of life (9, 10).

However, if the patient was diagnosed early and prompt surgical therapeutic management of the descending aortic malformation was performed, aneurysmal formation and rupture detected in the present case could have been averted. Thus, we appeal to local healthcare authorities to organize related medical staff training to strengthen awareness and improve physician's diagnostic skills. Finally, professional and public medical education concerning CoA is undoubtfully essential and should be emphasized in developing countries.

## Data Availability Statement

The raw data supporting the conclusions of this article will be made available by the authors, without undue reservation.

## Ethics Statement

The studies involving human participants were reviewed and approved by the Ethics Committee of the Second Xiangya Hospital of Central South University. Written informed consent to participate in this study was provided by the participants' legal guardian/next of kin. Written informed consent was obtained from the participants' legal guardian(s) for the publication of this case report.

## Author Contributions

KQ and CF drafted the manuscript. CF and JY designed the study. KQ, CI, CF, and JY revised the manuscript. KQ, MT, and JY were responsible for the collection of data or analysis. All authors read and approved the final manuscript.

## Funding

This work was supported by the Key Project of Science and Technology of Hunan Province (Grant No. 2020SK53420 to JY).

## Conflict of Interest

The authors declare that the research was conducted in the absence of any commercial or financial relationships that could be construed as a potential conflict of interest.

## Publisher's Note

All claims expressed in this article are solely those of the authors and do not necessarily represent those of their affiliated organizations, or those of the publisher, the editors and the reviewers. Any product that may be evaluated in this article, or claim that may be made by its manufacturer, is not guaranteed or endorsed by the publisher.

## Abbreviations

CoA: Coarctation of the aorta
CT: Computed tomography
CTA: Computed tomography angiography.
